# Perceived indoor annoyances at home and risk of incident depression: A Danish register-based cohort study, 2000–2018

**DOI:** 10.1097/EE9.0000000000000380

**Published:** 2025-03-26

**Authors:** Anne Marie Kirkegaard, Stine Kloster, Michael Davidsen, Anne Illemann Christensen, Klaus Martiny, Carlo Volf, Niss Skov Nielsen, Annette Kjær Ersbøll, Lars Gunnarsen

**Affiliations:** a Department of the Built Environment, Aalborg University, Copenhagen, Denmark; b National Institute of Public Health, University of Southern Denmark, Copenhagen, Denmark; c New Interventions in Depression, Mental Health Centre Copenhagen, Copenhagen, Denmark; d Department of Clinical Medicine, University of Copenhagen, Denmark; e Centre for Health Research, Zealand University Hospital, Nykoebing F., Denmark

**Keywords:** Perceived annoyance, Indoor environment, Environmental epidemiology, Depression, Cohort study

## Abstract

**Background:**

Exposures from the indoor environment can cause multiple annoyances that might increase the risk of depression. This study examines the association between perceived indoor annoyances at home and incident depression.

**Methods:**

This cohort study is based on data from 16,688 individuals (aged ≥16 years) who participated in the Danish Health and Morbidity Survey in the year 2000. Perceived levels of annoyances (few, moderate, and many) were based on information on perceived noise, low light levels, odor, and thermal discomfort in the home environment. Individuals were followed up to 19 years after inclusion through national registries. A generalized linear model with a Poisson distribution analyzed associations between perceived annoyances and incident rates of depression. Incidence rate ratios were adjusted for age, sex, educational level, cohabitation status, smoking status, years of residence at baseline, interview season, and calendar year.

**Results:**

Of the eligible 15,166 individuals, a total of 2,139 developed depression during the follow-up period. The incident rate of depression per 10,000 person-years was 241, 171, and 154 for many, moderate, and few perceived annoyances in the indoor environment at home. Individuals with many and moderate perceived annoyances had an adjusted incidence rate ratio of 1.56 (95% confidence interval [CI] = 1.28, 1.87) and 1.14 (95% CI = 0.94, 1.36) for developing depression compared with individuals with few perceived annoyances.

**Conclusion:**

The results show that individuals with moderate and many perceived annoyances in the indoor environment at home had a higher incidence rate of depression than individuals with few perceived annoyances.

**What this study adds**

This study adds to the limited literature on how indoor environmental annoyances due to noise, low light, odors, and thermal discomfort affect the risk of depression. We investigated the association between a combined exposure of perceived annoyances and incident depression in a cohort of 15,166 individuals followed for up to 19 years via Danish registries. The findings demonstrate that individuals who perceive moderate and many annoyances have a higher incidence rate of depression compared to those with fewer perceived annoyances.

## Background

Depression is a severe medical illness, and one in seven individuals in high-income countries will experience it during their lifetime.^1,2^ Depression negatively impacts the quality and length of life,^2^ and globally, depressive disorders are among the leading causes of disease burden.^3^ The well-known risk factors for depression include younger age, female sex,^2,4^ marital disruption,^2,5^ low income, short education,^1,4^ medical illness, early trauma, and adverse life events.^6^ The set of causes for depression is complex and partly unknown, although determined by genetics,^4,7^ biochemistry,^7^ psychiatric and physical comorbidity, and social and environmental factors.^4^ After the first episode of depression, the risk of recurrent depression is significantly increased.^8,9^ Annoyances in the indoor environment have been associated with depression in cross-sectional studies.^10–16^ While cohort and longitudinal studies with a large study size may not be sufficient to prove causality, they are valuable for better ruling out alternative explanations and proving stronger evidence for potential associations.

Humans in high-income countries spend approximately 90% of their time in indoor spaces^17^ and are constantly exposed to environmental factors that may lead to perceived annoyance.

Most studies investigating the impact of annoyances on depression investigate noise annoyances and find that it increases the risk of depression.^10–15^ Few studies have examined other annoyances,^12,13,16^ so the association between these annoyances and depression is not well understood. A recent study found that subjective evaluations of the home environment, such as better protection from disturbing light at night, more daylight entering the home, and perceived quality of the window views, were significantly associated with lower levels of depression.^18^ Others have associated lack of daylight or lack of a window with multiple adverse mental health outcomes (e.g., depression) and suggested that natural light may have antidepressant effects.^16,19,20^ Odor annoyance^21,22^ and perceived temperature^23,24^ have also been found to negatively impact health, yet no studies have, to our knowledge, investigated their association with depression. Lastly, a recent meta-analysis concluded that highly noise-annoyed individuals had a higher risk of depression.^14^

An assessment of multiple annoyances may reflect the environment more optimally than a single condition. Occupants are exposed to all indoor environmental parameters simultaneously. Therefore, their perception of the indoor environment is most likely a combination of different environmental factors. To our knowledge, no previous studies have investigated the impact of multiple annoyances from perceived thermal discomfort, low indoor light levels, odor, and noise simultaneously. However, one previous study considered multiple sources of noise^10^ and another study, multiple sources of odor.^13^ Yet, both studies examined multiple sources of annoyances within the same aspect of the environment, that is, not any mixed exposure, such as noise and odor at the same time. There is a known interaction between occupants, buildings, and indoor environment quality.^25^ Since perceived annoyances related to several sources tend to cluster,^26,27^ it can be problematic to draw conclusions based on one annoyance indication alone, such as in previous research. Thus, the perception of one kind of annoyance may also be correlated with the perception of other annoyances, that is, multiple annoyances may co-occur and originate from the same source.^13^

Several mechanisms have been proposed to explain the observed associations.^28^ For instance, annoyances might be considered a proxy for dissatisfaction and distress associated with actual exposure,^14,29–33^ thus acting as a mediator between the source (e.g., actual noise level) and health outcome.^14,33–36^ The exact source, for example, air pollution, could, besides leading to annoyance, also potentially lead to depression through biological pathways,^28,37,38^ and perceived air pollution may also lead to cognitive stress and symptoms of ill health.^28^ Furthermore, annoyances might change the annoyed individual’s behavior, such as limiting physical or social activities, which might lead to mental ill health.^28^

This study aimed to examine the association between perceived annoyances in the indoor environment at home and the incidence rate (IR) of depression.

## Materials and methods

### Study design and setting

We conducted a cohort study with up to 19 years of follow-up based on cross-sectional data from the Danish Health and Morbidity Survey in the year 2000. The population invited to the Danish Health and Morbidity Survey was a nationwide representative random sample of 22,486 individuals, where a total of 16,688 individuals participated (response rate 74%). The characteristics of the Danish Health and Morbidity Survey design have been described in detail elsewhere.^39,40^

All Danish residents are assigned a unique personal identification number that allows individual-level information to be linked between data sources.^41^ Based on the unique personal identification numbers, we could follow participants in national registries.

### Population

This study included Danish residents aged ≥16 years with no prior episodes of depression. Individuals were excluded if they had missing residential addresses or could not be linked to registries.

The study population was followed up via Danish healthcare and population registries. Risk time started on the interview date or 6 months after moving in, whichever occurred last, and was calculated until a depression, death, emigration, 6 months after moving out, or the end of the study on 31 December 2018, whichever came first. The cutoff at 6 months was determined to ensure a depression could be ascribed to the indoor environment for the respondent and not previous or subsequent housing.

### Data sources

The Danish Civil Personal Register contains information on all individuals residing in Denmark since 1968.^41^

The Danish National Patient Registry contains information on somatic inpatients since 1977.^42^ Since 1995, the Danish National Patient Registry has included information on all outpatient activities, emergency room contacts, and activities in psychiatric wards,^42^ The Psychiatric Central Register is an integrated part of the Danish National Patient Register, and since 1969, it has covered every psychiatric admission, and, since 1995, outpatient treatment and emergency room contacts.^43^ The diagnoses were coded according to the 8th revision of the International Classification of Diseases (ICD-8) from 1977 to 1994, and after that, according to the 10th revision (ICD-10).^42^

The Danish National Prescription Registry holds individual-level data on all prescriptions filled by Danish residents at community pharmacies since 1995.^44^ The drugs were coded according to the Anatomical Therapeutic Chemical (ATC) classification system.

The Population’s Education Register contains data on individuals’ highest completed education with information available for cohorts born after 1945.^45^

The Building and Housing Register contains information on the total building stock and individuals’ housing conditions since 1977, yet considered consistent and comparable since 1994.^46^ All unique housing units are assigned a unique number, which can be linked to other databases that include the unique personal identification number of residents.^46^

The Danish Health and Morbidity Survey in the year 2000 was conducted as a personal interview in the respondents’ home.^40^ Part of the survey was dedicated to questions about the indoor environment and perceived annoyances. Further self-reported information was collected, such as smoking status and self-rated health (details are reported elsewhere^40^).

### Depression

Incident depression (i.e., the first episode) was identified by linking individuals with the Danish National Patient Registry and the Danish National Prescription Registry. Depression was defined based on having either a hospital contact or a redeemed prescription at pharmacies. Depression included primary, secondary, and additional diagnoses of the ICD-10 codes F32 and F33 (including subcategories). Antidepressant medication included ATC code N06A (including subcategories) except N06AX12. To identify depression before enrollment, we included the corresponding ICD-8 codes 296.0, 296.2, 296.8, 298, 300.4, 311, and 313.1, as individuals diagnosed with depression before enrollment were not included.

### Perceived annoyances

Information on perceived annoyances in the indoor home environment included thermal discomfort, low indoor light levels, odor or stuffy air, and noise (see Additional file 1; http://links.lww.com/EE/A338). Individuals from the Danish Health and Morbidity Survey were asked, “Within the last 14 days, have you been annoyed by the following condition?.” Twelve specific conditions were listed, for which individuals rated each on a scale of 0 (not annoyed), 1 (a little annoyed), and 2 (very annoyed). For statistical purposes, answers were categorized as not annoyed (0) or annoyed (1, 2). Additionally, individuals were asked, “Do you live next to a trafficked road?” (no/yes) to further assess perceived annoyances.

The 13 variables related to perceived indoor annoyances in the home environment were integrated into a Latent Class Analysis, which assigned individuals to a group based on the highest probability of belonging due to similarity to others in terms of their reported annoyances. This process resulted in an ordinal variable with three levels: Few, moderate, and many annoyances. The final model, which included three classes, was chosen because it had the lowest Bayesian information criterion and log-likelihood. Detailed information on the methods for assessing perceived annoyances is available elsewhere.^26^

### Confounders and other variables

We determined potential confounding variables using a directed acyclic graph (See Additional file 2; http://links.lww.com/EE/A338) created with the DAGitty web application.^47^ The minimal sufficient adjustment set included 78 variations to estimate the effect of annoyances on depression. Since we could not access all variables, we selected the adjustment set where the accessible variables were considered most valid and with the fewest missing variables (i.e., missing objective sound level and temperature). Potential confounding variables were age (5-year intervals), sex, educational level (mandatory, secondary/vocational, medium/long), cohabitation status at baseline (married/cohabitating, living alone), and baseline smoking status (current, previous/never). We further adjusted for years lived in residence at baseline (<3, 3–7, 8–12, 13–20, and ≥21), calendar year (<2002, 2002–2004, 2005–2007, 2008–2010, 2011–2013, and ≥2014), and season of enrollment (spring, summer, autumn, and winter).

The highest completed education was updated yearly and categorized according to the International Standard Classification of Education System (ISCED).^48^ Furthermore, it was aggregated into three groups: mandatory (ISCED levels 1–2), secondary or vocational (ISCED levels 3–4), and medium or long education (ISCED levels 5–8).

Further variables were included to describe the population in more detail and to include in sensitivity analyses. This included information on country of origin (Danish or non-Danish), self-rated health (very good, good, fair, poor, or very poor), house type (detached house, semi-detached and terrace houses, apartments, farms, other house types, or missing), urbanization (<200, 200–4999, 5000–49999, ≥50000 residents, or missing), residential density (<40, 40–79, and ≥80 square meters/resident, or missing), and number of residents in the household (1, 2, 3, 4, or ≥5).

### Statistical methods

A sample size calculation was performed to determine the minimum detectable incidence rate ratio (IRR) for depression (see Additional file 3; http://links.lww.com/EE/A338).

Baseline data were analyzed by frequencies and percentages, and the mean follow-up time was calculated for the total population and each level of annoyance. Furthermore, we calculated the number of depressions, person-years at risk, and the incidence rate per 10,000 person-years at risk.

The association between annoyances and the incidence rate of depression was evaluated by a time-to-event analysis. We applied a generalized linear model with a Poisson distribution of the number of depressions and a logarithmic transformation of risk time as the offset value.^49^ Risk time was split by age and calendar year using SplitMulti in R from the PopEpi package after having constructed a Lexis object in R using the Epi package.^50^ We split the risk time as the model assumption requires the IR of depression to be constant within each time interval. To account for nonresponses, we used weights computed by Statistics Denmark based on information on age, ethnicity, and income.^51,52^ We estimated the IRRs of depression with 95% confidence intervals (CI) based on the level of perceived annoyances in a crude and adjusted model. All analyses were performed in R version 4.1.3.

### Sensitivity analyses

We performed four sensitivity analyses to evaluate the definition of depression by repeating the main analysis with other depression definitions (see Additional files 4 and 5; http://links.lww.com/EE/A338.

In the main analysis, the prominent annoyances were slightly different for individuals with few, moderate, and many annoyances. Therefore, two sensitivity analyses tested whether there was an exposure-response relation between the accumulated number of annoyances and depression.

Previous studies have shown an association between noise annoyance and depression,^14^ therefore, to preclude that noise annoyance was responsible for the association found in the main analysis, we performed four additional analyses. We tested whether perceived annoyances due to noise, thermal discomfort, low light levels, odor, and stuffy air, respectively, were associated with depression.

Finally, we restricted the study population by excluding individuals with poor, very poor, or missing self-rated health at baseline, as poor self-rated health may be linked to undiagnosed depression.^53,54^ The methods are described in Additional files 4 and 6; http://links.lww.com/EE/A338.

## Results

A total of 15,166 individuals were eligible for this register-based cohort study (details in Figure). Individuals with many annoyances were more likely than those with few annoyances to be women, younger than 30 years old, married/cohabiting, nonsmokers, have a short education, live in apartments, and in city with ≥50,000 residents (Table 1). Moreover, individuals with many annoyances tended to have less than 40 square meters per resident, have lived less than 3 years in the residence, and were enrolled during the winter. Among individuals with many annoyances, 28.8% had very good self-reported health compared with 36.0% and 37.9% among individuals with moderate and few annoyances, respectively (Table 1).

**Table 1.** Baseline characteristics of individuals (total n = 15,166) by level of perceived annoyances in the indoor environment

		Annoyances, n (%)		
	*n*	Few (n = 13,483)	Moderate (n = 885)	Many (n = 798)
Demographic characteristics				
Sex				
Male	7,663	6,850 (50.8)	467 (52.8)	346 (43.4)
Female	7,503	6,633 (49.2)	418 (47.2)	452 (56.6)
Age group, years				
<25	2,138	1,785 (13.2)	138 (15.6)	215 (26.9)
25–29	1,311	1,060 (7.9)	105 (11.9)	146 (18.3)
30–34	1,369	1,180 (8.8)	84 (9.5)	105 (13.2)
35–39	1,387	1,224 (9.1)	90 (10.2)	73 (9.1)
40–44	1,377	1,238 (9.2)	91 (10.3)	48 (6)
45–49	1,394	1,266 (9.4)	77 (8.7)	51 (6.4)
50–54	1,457	1,343 (10)	75 (8.5)	39 (4.9)
55–59	1,263	1,174 (8.7)	64 (7.2)	25 (3.1)
60–64	922	847 (6.3)	45 (5.1)	30 (3.8)
65–69	758	698 (5.2)	38 (4.3)	22 (2.8)
70–74	642	605 (4.5)	24 (2.7)	13 (1.6)
75–79	527	484 (3.6)	27 (3.1)	16 (2)
80–84	354	328 (2.4)	17 (1.9)	9 (1.1)
≥85	267	251 (1.9)	10 (1.1)	6 (0.8)
Highest attained education^a^				
Mandatory education	5,681	5,076 (37.7)	287 (32.5)	318 (39.8)
Secondary or vocational education	6,425	5,676 (42.1)	408 (46.2)	341 (42.7)
Medium or long education	305	2,722 (20.2)	189 (21.4)	139 (17.4)
Smoking status				
Smoker	5,476	4,766 (35.3)	327 (36.9)	383 (48)
Nonsmoker	9,671	8,698 (64.5)	558 (63.1)	415 (52)
Missing	19	19 (0.1)	0 (0)	0 (0)
Country of origin				
Danish	14,869	13,223 (98.1)	867 (98)	779 (97.6)
Non-Danish^b^	297	260 (1.9)	18 (2)	19 (2.4)
Cohabitation status				
Living alone	4,767	4,116 (30.5)	298 (33.7)	353 (44.2)
Married/cohabiting	10,399	9,367 (69.5)	587 (66.3)	445 (55.8)
Self-rated health^c^				
Very good	565	5,101 (37.9)	319 (36)	230 (28.8)
Good	6,604	5,841 (43.4)	418 (47.2)	345 (43.2)
Fair	2,212	1,929 (14.3)	111 (12.5)	172 (21.6)
Poor	537	468 (3.5)	29 (3.3)	40 (5)
Very poor	152	133 (1)	8 (0.9)	11 (1.4)
Household and environmental characteristics				
House type				
Detached house	7,878	7,278 (54)	384 (43.4)	216 (27.1)
Semi-detached and terrace houses	251	2,223 (16.5)	136 (15.4)	151 (18.9)
Apartments	3,067	2,420 (17.9)	310 (35)	337 (42.2)
Farms	1,267	1,184 (8.8)	29 (3.3)	54 (6.8)
Other house types	350	296 (2.2)	21 (2.4)	33 (4.1)
Missing	94	82 (0.6)	5 (0.6)	7 (0.9)
Year of house construction				
<1960	7,056	6,005 (45)	568 (64)	483 (61)
1960–1978	5,089	4,671 (35)	217 (25)	201 (25)
≥1978	2,429	2,282 (17)	67 (8)	80 (10)
Missing	592	525 (4)	33 (4)	34 (4)
Urbanization				
<200 residents (rural)	2,437	2,256 (16.7)	81 (9.2)	100 (12.5)
200–4,999 residents	3,841	3,533 (26.2)	182 (20.6)	126 (15.8)
5,000–49,999 residents	4,512	4,030 (29.9)	255 (28.8)	227 (28.4)
≥50,000 residents	3,828	3,175 (23.5)	336 (38)	317 (39.7)
Missing	548	489 (3.6)	31 (3.5)	28 (3.5)
Residential density, square meters/resident				
<40	5,209	4,462 (33.1)	347 (39.2)	400 (50.1)
40–79	6,915	6,263 (46.5)	364 (41.1)	288 (36.1)
≥80	2,448	2,231 (16.5)	141 (15.9)	76 (9.5)
Missing	594	527 (3.9)	33 (3.7)	34 (4.3)
Number of residents in household				
1	2,848	2,470 (18.3)	194 (21.9)	184 (23.1)
2	578	5,138 (38.1)	367 (41.5)	275 (34.5)
3	2,622	2,347 (17.4)	133 (15)	142 (17.8)
4	2,579	2,315 (17.2)	129 (14.6)	135 (16.9)
≥5	1,337	1,213 (9)	62 (7)	62 (7.8)
Years lived in residence				
<3	4,429	3,709 (27.5)	349 (39.4)	371 (46.5)
3–7	3,321	2,920 (21.7)	197 (22.3)	204 (25.6)
8–12	1,834	1,667 (12.4)	85 (9.6)	82 (10.3)
13–20	2,386	2,212 (16.4)	93 (10.5)	81 (10.2)
≥21	3,196	2,975 (22.1)	161 (18.2)	60 (7.5)
Interview details				
Season of enrolment				
Spring	3,631	3,221 (23.9)	205 (23.2)	205 (25.7)
Summer	2,543	2,278 (16.9)	157 (17.7)	108 (13.5)
Autumn	6,148	5,481 (40.7)	386 (43.6)	281 (35.2)
Winter	2,844	2,503 (18.6)	137 (15.5)	204 (25.6)

Values are numbers and percentages in columns.

a Missing n = 10.

b Non-Danish consists of immigrants of western/nonwestern origin and descendants.

c Missing n = 2.

**Figure.**
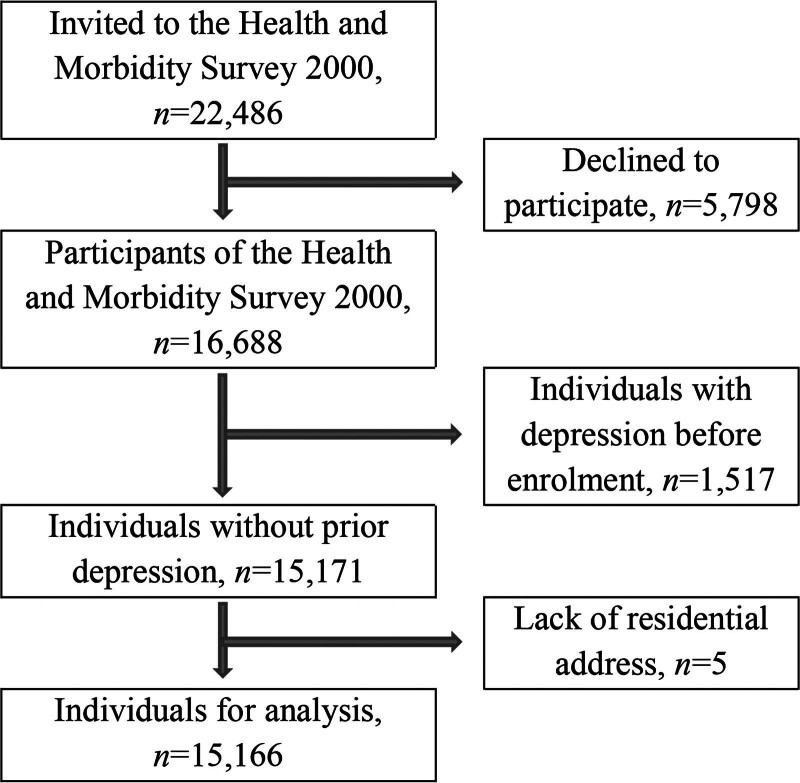
Flow diagram of cohort selection from the Danish Health and Morbidity Survey 2000.

A total of 2,139 (14.1%) developed depression during the follow-up period. The mean follow-up was 8.9 years, providing an IR of depression of 158 per 10,000 person-years at risk (few: 154, moderate: 171, and many annoyances: 241) (Table 2).

**Table 2.**
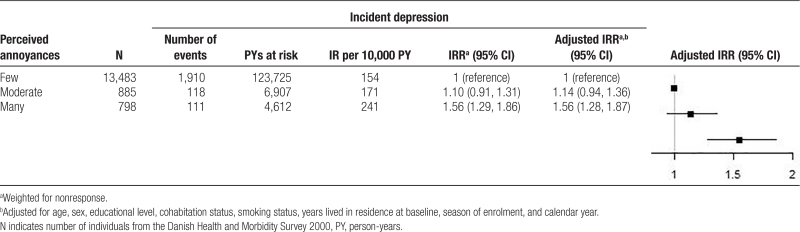
Poisson regression of rates of depression

The IRR of depression was highest for individuals with many annoyances with an adjusted IRR of 1.56 (95% confidence interval [CI] = 1.28, 1.87) (Table 2). Among individuals who perceived moderate annoyances, the adjusted IRR was 1.14 (95% CI = 0.94, 1.36) compared to individuals with few annoyances.

Sensitivity analyses showed similar patterns as the main analysis when restricting the depression definition to a minimum of two medicine prescriptions within a period of 12 months (sensitivity analyses 1 in Additional file 7; http://links.lww.com/EE/A338). Also, when restricting ATC codes to include indication codes against and for prevention of depression (sensitivity analyses 3 in Additional file 7; http://links.lww.com/EE/A338). Lastly, also when expanding the main definition with diagnoses of anxiety disorders (sensitivity analyses 4 in Additional file 7; http://links.lww.com/EE/A338). Only evaluating diagnoses from hospital contacts strengthened the association as the adjusted IRR was 1.53 (95% CI = 0.95, 2.33) and 1.97 (95% CI = 1.21, 3.04) for individuals with moderate and many annoyances compared with few annoyances, yet IRs were much smaller than in the main results (sensitivity analysis 2 in Additional file 7; http://links.lww.com/EE/A338).

Perceiving three or more annoyances showed significantly higher IRRs for depression compared with no annoyances (adjusted IRR = 1.70, 95% CI = 1.42, 2.03), where the analysis showed no significant difference for one or two annoyances (adjusted IRR = 1.08, 95% CI = 0.99, 1.19) compared with no annoyances (sensitivity analysis 5 in Additional file 7; http://links.lww.com/EE/A338). The same trend was found when evaluating a greater number of annoyances in sensitivity analysis 6. The main analysis was repeated for each type of annoyance. An increasing number of noise annoyances was associated with a higher IR of depression with adjusted IRR of depression at 2.02 (95% CI = 1.23, 3.08) for three or more annoyances compared with no annoyances (sensitivity analysis 7 in Additional file 7; http://links.lww.com/EE/A338). Two annoyances due to thermal discomfort were associated with higher IRR of depression (adjusted IRR = 1.75, 95% CI = 1.33, 2.26) compared to no annoyances, yet neither one nor three annoyances significantly increased the IRR (sensitivity analysis 8 in Additional file 7; http://links.lww.com/EE/A338). Perceived annoyances due to low levels of light and odor and stuffy air, respectively, were associated with depression with an adjusted IRR of 1.73 (95% CI = 1.24, 2.32) and 1.47 (95% CI = 1.18, 1.81) compared with no annoyances (sensitivity analysis 9 and 10 in Additional file 7; http://links.lww.com/EE/A338).

Restricting the study population to individuals with fair, good, and very good self-rated health increased the adjusted IRR to a level of significance for individuals with moderate annoyance (1.22; 95% CI = 1.00, 1.46) compared with moderate annoyance in the main analysis. However, for individuals with many annoyances, the adjusted IRR did not differ in the sensitivity analysis compared to the findings in the main analysis (sensitivity analysis 11 in Additional file 7; http://links.lww.com/EE/A338).

## Discussion

We found that a higher level of annoyance was associated with a higher incidence rate of depression.

Our results are consistent with previous findings,^10–16,18^ indicating the potentially harmful impact of annoyances on depression. To the best of our knowledge, this study was the first to apply an exposure that simultaneously reflected multiple sensory elements of the indoor environment when investigating the association with depression. Therefore, a comparison with previous studies should be done with caution. A meta-analysis of eight studies investigating noise annoyance found the pooled odds ratio of depression was 1.23 (95% CI = 1.03, 1.48) for highly noise-annoyed individuals.^14^ A brief review concluded that noise annoyance was positively correlated with mental health issues, depressive symptoms, and antidepressant use.^15^ Additional studies have reported consistent findings.^10,11,13^ Moreover, higher levels of odor annoyance from agriculture and other sources, respectively, significantly increased psychological distress.^13^ Individuals who were visually annoyed by wind turbines had a significantly higher risk of depression than before the wind turbines were set up, whereas no significant change was found for individuals who were annoyed by noise from wind turbines.^12^ One study found that individuals who reported inadequate natural light in their dwelling had an adjusted odds ratio of depression of 1.3 (95% CI = 1.1, 1.6) compared with those satisfied with their dwelling’s light.^16^ Likewise, others have demonstrated that lack of natural light was associated with high cortisol levels and lower melatonin levels at night, and these, in turn, were associated with depressive symptoms and poor sleep quality.^55^ To our knowledge, no previous studies have investigated the association between perceived thermal annoyances and depression. However, a systematic review found that high indoor temperatures affected human health; for example, core symptoms of schizophrenia and dementia were significantly exacerbated by indoor heat.^56^ Another study found that lower temperatures were associated with an increased risk of depression.^57^

This study found an association between perceived annoyance and incident depression that several factors may explain. Annoyance might lead to increased interpersonal distance and social isolation, thus leading to mental ill health.^28^ Likewise, annoyances from outdoor sources, such as noise from trafficked roads or perceived air pollution, might limit outdoor physical activity, social interactions, or sleep quality, thereby decreasing mental health.^24,28^

Another explanation is that the physical aspects of the environment may influence the risk of depression, particularly among genetically susceptible individuals.^4^ Some individuals are more sensitive and affected by noise, and one study found this aggregated within families, which indicates that noise sensitivity might be a genetic component.^58,59^ The heritability of noise sensitivity was estimated to be 36%.^60^ Previous studies have reported that only up to one-third of the variance in noise annoyance is explained by personality, social factors, and other factors, such as the timing of occurrence and the possibility of escaping it.^13,29,61^ Additionally, one-third may be explained by noise levels.^13,61^ The remaining one-third remains unexplained.^29,61^ Berkers et al^13^ suggested a similar assumption was reasonable for odor; thus, up to one-third of the variance in odor annoyance might be explained by odor levels. However, it remains uncertain how much of the variance is explained by the actual temperature and light levels. Perceived annoyance towards odors, low light levels, and temperature might also be influenced by sensitivity and genetic components. Moreover, the impact of annoyances might be increased by repeated disturbances, leading to an emotional or attitudinal response such as anger or negative evaluations of the source.^32^

The finding of an association may also be explained by house quality and occupants’ health status. Houses where residents perceive annoyances might be houses where residents were more socially and economically challenged, as the house (e.g., disrepair and location) made it less appealing to live in. Thus, the house price was reduced. It is well-known that individuals with socio-economic deprivation might have poorer general health^62,63^ and live in lower-quality houses.^64–66^

The underlying biological mechanisms that might link annoyances with depression are unclear. Still, annoyances are believed to trigger adverse emotional reactions, including the release of stress hormones,^13,14,67,68^ which disrupt hormonal rhythms via activation of the hypothalamic-pituitary-adrenal axis.^14,68^ Dysregulation of the hypothalamic-pituitary-adrenal axis is strongly associated with depression.^14,69,70^ Furthermore, studies have shown a direct pathway from the source of annoyances to a reduction in mental health, where the nervous system is negatively affected by physical arousal and activation of stress hormones.^13^ For example, lack of adequate daylight in the built environment influences the circadian system and, thus, the risk of depression.^71^ Another biological pathway from annoyance to depression may be through air pollution, as previous studies have found that some air contaminants might influence neurobehavioral functions by entering the brain directly via the olfactory system.^28,38^ or by promoting pro-inflammatory cytokines that penetrate the blood-brain barrier.^28,37^

### Strengths and limitations

The strengths of our study include the cohort design with the exposure assessment preceding the outcome, unique self-reported information collected at enrollment (e.g., perceived annoyances and smoking status), virtually complete information on potential confounders and residential address, and up to 19 years of follow-up. Furthermore, depression diagnoses in the Danish National Patient Registry are considered of high quality and validated.^43,72^ Severe mental disorders are almost completely registered; however, mild to moderate mental disorders are not fully registered.^43^ Data from the Danish National Prescription Registry are also considered complete and valid because of the systems used to minimize the risk of data entry errors and because pharmacies receive a financial incentive for complete registration of purchases.^44^

The results proved robust across different definitions of depression, as similar findings emerged in the sensitivity analyses. The results were moreover robust regarding annoyance levels; further sensitivity analyses showed that the number of annoyances in total and for each type individually increased the IR of depression compared to no annoyances. Therefore, it is plausible to assume that perceived annoyances from noise, low light levels, odor, stuffy air, and thermal discomfort were responsible for the associations observed in the main analysis. Moreover, the response rate was high (74%), and we followed participants through registries with low drop-out and accounted for nonresponses.

Our study also has some limitations. First, perceived annoyance was based on the baseline responses. Variations in the perception of annoyances due to personal, social, or external changes in the environment^29,73^ may have occurred over time, which could not be accounted for. However, we were able to update some confounders, such as age and educational level.

A second limitation was the lack of information on past depressive episodes, as before 1995, only somatic inpatients were registered (dating back to 1969).^42^ Information on prescriptions was absent until 1995, and information on indication codes until May 2004.^44,74^ Therefore, we risked including individuals with a prior depression. The risk of recurrence is substantially higher in the first 5 years.^75,76^ Thus, by having complete data for 5 years before enrollment, we were able to exclude individuals with the highest risk of recurrent depression. Furthermore, antidepressants may be linked to diseases other than depression, such as anxiety disorders, yet the most common cause is depression.^4,77^ Information on prescriptions were available only from community pharmacies,^44^ and not hospitals or clinics. Individuals with hospital-issued prescriptions are likely recorded due to the hospital contact or when collecting prescriptions at the community pharmacy posthospitalization.

We presented a detailed directed acyclic graph illustrating that objective measures of sound and temperature should have been included as confounders in the multivariable analyses. However, due to the unavailability of this data, we cannot assess the extent to which their inclusion would have impacted the findings. While adjusting for confounding factors typically reduces observed differences in outcomes, this is not always the case.^78^ Berkers et al^13^ controlled for a range of confounders, including modeled indicator variables for noise and odor, and still found significant associations between annoyances on depression.

There was a possible risk of reverse causality as a result of a “social drift” where individuals more prone to depression lived in lower-quality housing leading to more annoyances. However, the prospective cohort design and confounder adjustments addressed the direction and relationship between variables.

### Implications

In recent years, depression has become one of the greatest disease burdens worldwide.^3^ It is possible that reducing annoyances in indoor environments could play a role in preventing some depression diagnoses. However, more research is needed, for example, on the pathways, as few studies have investigated this potential link between perceived annoyances and depression. While the findings from the present study suggest a possible connection with individuals perceiving many annoyances having a 1.56 times higher risk of depression compared with individuals with few annoyances.

Perceived annoyances have been linked to several adverse health effects.^14,79,80^ Thus, dwelling designers and renovators should aim to reduce annoyance. A survey covering perceived annoyances among residents might be beneficial in identifying individuals at risk and initiating targeted interventions to reduce depression.

## Conclusions

The results showed that perceived indoor annoyances at home were associated with depression. The association was strongest for individuals with many indoor annoyances and thereafter a moderate perception of indoor annoyances compared with few perceived indoor annoyances. The present study was the first to combine perceived annoyances that reflected multiple aspects of the indoor environment simultaneously (i.e., perceived inadequate levels of light, noise, thermal discomfort, and odor and stuffy air) when examining the association with depression. Further research on exposures of perceived annoyances from several sources simultaneously is needed to elaborate these findings.

## Supplementary Material


