# Supplementary material for: Perceived indoor annoyances at home and risk of incident depression: A Danish register-based cohort study, 2000–2018
Source: Environ Epidemiol. 2025 Mar 26;9(2):e380. doi: 10.1097/EE9.0000000000000380 (PMC11949297; doi:10.1097/EE9.0000000000000380)
Supplement: Supplementary file 1 [file ee9-9-e380-s001.pdf]

Supplementary Material

Additional file 1 Questions and distribution of perceived indoor annoyances

Perceived annoyances were assessed by the following question and the percentage of annoyed is provided for each condition.

**Have You, within the last 14 days, been annoyed by any of the following conditions in your home?**	**Not annoyed, %**	**Slightly annoyed, %**	**Very annoyed, %**
1) Too low/high temperatures	94.1	4.4	1.5
2) Draught	95.6	3.2	1.2
3) Draught along the floor	93.0	5.3	1.7
4) Odour or stuffy air	96.7	2.2	1.1
5) Traffic noise	93.8	4.1	2.1
6) Noise from installations (e.g. from drain, radiators, refrigerator)	97.2	1.9	0.9
7) Noise from neighbours	92.2	4.8	3.0
8) Noise from nearby industry	98.5	0.9	0.6
9) Infrasound or low-frequency sound	99.2	0.5	0.3
10) Vibration in building (e.g. from traffic)	97.7	1.5	0.8
11) Shock from static electricity	99.1	0.6	0.3
12) The dwelling was too dark	98.4	1.1	0.5

Percentages are retrieved from the Danish Health and Morbidity Survey 2000: Interviewskema med svarfordelinger [Interview questionnaire with distributions of answers] [1], page 39. Please note that the number of individuals differs from this study.

Furthermore, the question: "Do you live next to a trafficked road?" (no/yes)

Additional file 2

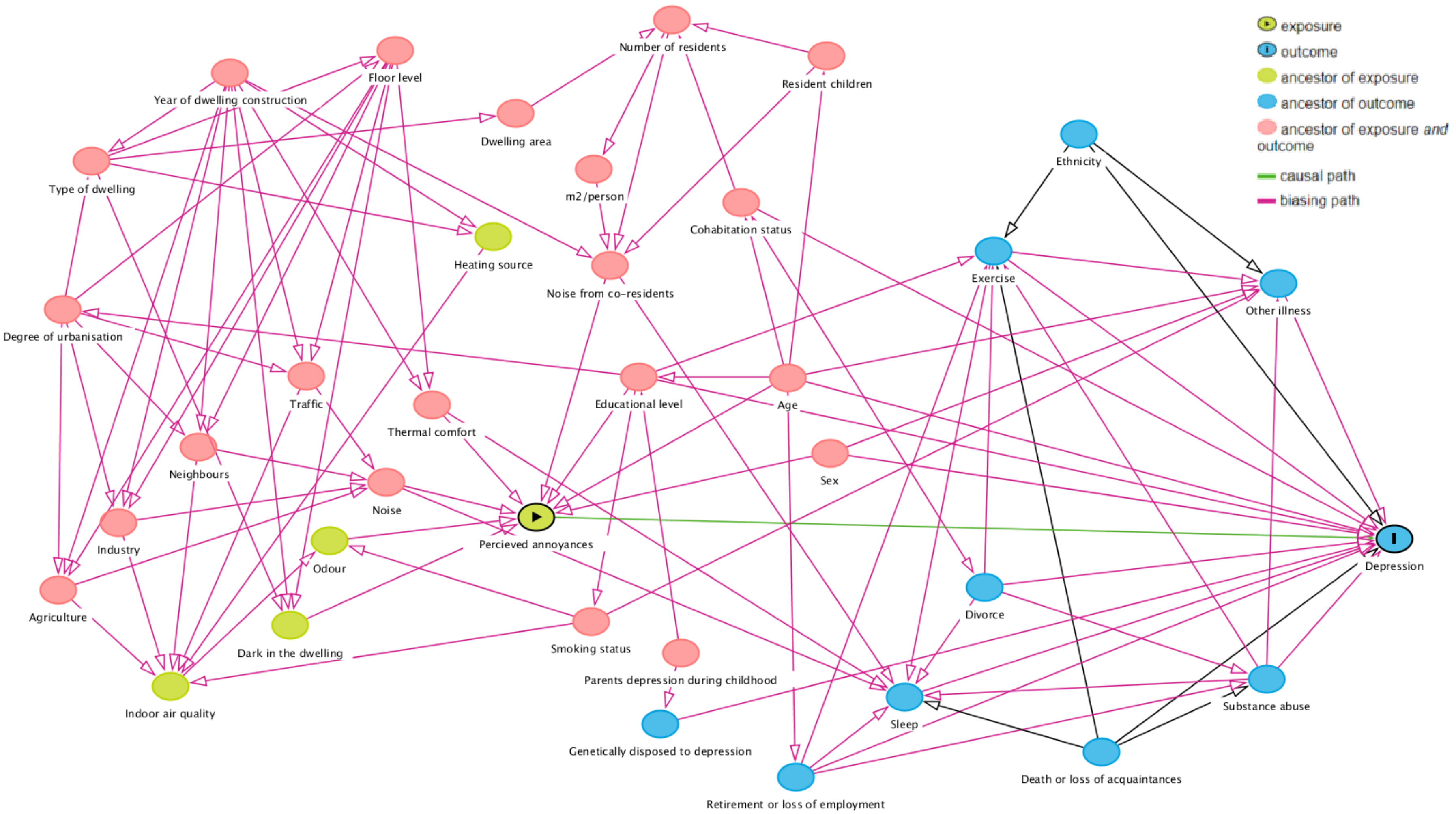

Supplemental Figure 1 Simplified Directed Acyclic Graph (DAG) highlighting variables of importance in the analysis of the association between percieved annoyances in the indoor environment at home and depression

Additional file 3 Sample size calculation

The minimum detectable incidence rate ratio (IRR) for depression was 1.41 comparing individuals with many annoyances to those with few annoyances.[2] This is based on the following assumptions: a study population of 14,400 individuals with few and many annoyances, 26.5% perceives annoyances,[3] an incidence rate (IR) of depression in the general population at 190.5 per 100,000 person-years,[4] on average 9 years follow-up, a 5% significance level, and a power of 80%.[2]

Additional file 4 Overview of sensitivity analyses

First, we restricted the depression criteria for ATC codes to a minimum of two prescriptions within a period of 12 months, while keeping the criteria for ICD codes. Secondly, we restricted the depression criteria to only include ICD-10 F32-32.9 diagnoses confirmed in the validity study by Bock et al. (2009). Thirdly, the main definition using the ICD codes remained, while prescriptions should include antidepressants (ATC codes N06A) with the indication code '168' ('against depression') or '270' ('for prevention of depression'). The fourth sensitivity analysis included anxiety (ICD-8 298.2; 300.0; 300.1; 300.2; 300.3; 300.4; 300.5; 300.6; 300.7; 300.8; 300.9; 307; 309.0-309.9; ICD-10 DF40-48) beside the main definition of depression, as the comorbidity of anxiety and depression is considerably influenced by diagnostic overlap.[5] The remaining sensitivity analyses had different annoyance exposures based on summery scores, i.e., counting the number of annoyances for each individual. The fifth and sixth sensitivity analysis used a summery score on all annoyances of 0, 1-2, and ≥3 annoyances, and 0, 1, 2, 3, and ≥4 annoyances, respectively. In a similar manner, sensitivity analysis seven to ten counted the number of annoyances within noise (0, 1, 2, and ≥3), thermal discomfort (0, 1, 2, and 3), low levels of light (0, and 1), and odour/stuffy air (0, and ≥1). The eleventh sensitivity analysis replicated the main analysis, but with the study population restricted to individuals who reported very good, good and fair self-reported health at baseline.

Additional file 5

Supplementary Table 1 ICD and ATC codes used to define incident depression in sensitivity analyses 1 to 4

	**ICD-10 codes** [a]	**ICD-8 codes**	**ATC codes**
Sensitivity analysis 1	F32 [b]	296.0; 296.2; 296.8; 298; 300.4; 311; 313.1	N06A [b] minus N06AX12 (bupropion). Minimum of two medicine prescriptions within a period of 12 months.
Sensitivity analysis 2	F32-32.9	296.0; 296.2; 296.8; 298; 300.4; 311; 313.1	-
Sensitivity analysis 3	F32 [b]	296.0; 296.2; 296.8; 298; 300.4; 311; 313.1	N06A [b] with the indication code '168' ('against depression') or '270' ('for prevention of depression')
Sensitivity analysis 4 (includes anxiety disorders)	F32 [b]<br>DF40-48	296.0; 296.2; 296.8; 298; 300.4; 311; 313.1<br>298.2; 300.0; 300.1; 300.2; 300.3; 300.4; 300.5; 300.6; 300.7; 300.8; 300.9; 307; 309.0-309.9	N06A [b] minus N06AX12 (bupropion)

ICD-10: International Statistical Classification of Diseases, Tenth Revision; ICD-8: International Classification of Diseases, 8th Revision; ATC: Anatomical Therapeutic Chemical; [a] Primary, secondary, and additional diagnoses; [b] Including subcategories.

N06AX12 is only prescribed for smoking cessation in Denmark.[6]

Additional file 6

Supplementary Table 2 Items used to define the exposure in sensitivity analyses 5 to 10

<table>
<tr><th></th><th>Exposure</th><th>Categories</th><th>Items [a]
Perceived annoyances within the past 14 days were assessed by asking individuals whether they had been annoyed by:</th></tr>
<tr><td>Sensitivity analysis 5</td><td>Number of all perceived annoyances</td><td>0, 1-2, ≥3</td><td rowspan="2">(1) Too low/high temperatures
(2) Draught
(3) Draught along the floor
(4) Odour or stuffy air
(5) Traffic noise
(6) Noise from installations (e.g. from drain, radiators, refrigerator)
(7) Noise from neighbours
(8) Noise from nearby industry
(9) Infrasound or low-frequency sound
(10) Vibration in building (e.g. from traffic)
(11) Shock from static electricity
(12) The dwelling was too dark</td></tr>
<tr><td>Sensitivity analysis 6</td><td>Number of all perceived annoyances</td><td>0, 1, 2, 3, ≥4</td></tr>
<tr><td>Sensitivity analysis 7</td><td>Number of perceived annoyances due to noise</td><td>0, 1, 2, ≥3</td><td>(1) Traffic noise
(2) Noise from installations (e.g. from drain, radiators, refrigerator)
(3) Noise from neighbours
(4) Noise from nearby industry
(5) Infrasound or low-frequency sound
(6) Vibration in building (e.g. from traffic)</td></tr>
<tr><td>Sensitivity analysis 8</td><td>Number of perceived annoyances due to thermal discomfort</td><td>0, 1, 2, 3</td><td>(1) Too low/high temperatures
(2) Draught
(3) Draught along the floor</td></tr>
<tr><td>Sensitivity analysis 9</td><td>Number of perceived annoyances due to low levels of light</td><td>0, 1</td><td>(1) The dwelling was too dark</td></tr>
<tr><td>Sensitivity analysis 10</td><td>Number of perceived annoyances due to odour or stuffy air</td><td>0, ≥1</td><td>(1) Odour or stuffy air
(2) Shock from static electricity</td></tr>
</table>

[a] Responses to each item were characterised 0 (not annoyed) or 1 (yes, slightly annoyed / yes, very annoyed) and summarized, so individuals could be allocated to a category.

Additional file 7

Supplementary Table 3 Poisson regression of rates of depression among individuals from the Danish Health and Morbidity Survey 2000

Perceived annoyances	N	Incidence depression				
		**Number of events**	**PYs at risk**	**IR per 10,000 PY**	**IRR [a] (95% CI)**	**Adjusted IRR [a,] (95% CI)**
**Sensitivity analysis 1 [b]**						
**Few**	13,769	1,612	128,272	125	1 (reference)	1 (reference)
**Moderate**	910	97	7,180	135	1.07 (0.87, 1.30)	1.12 (0.91, 1.35)
**Many**	817	93	4,868	191	1.51 (1.23, 1.84)	1.51 (1.22, 1.84)
**Sensitivity analysis 2 [b]**						
**Few**	14,697	238	144,919	16	1 (reference)	1 (reference)
**Moderate**	972	18	8,102	22	1.44 (0.90, 2.20)	1.53 (0.95, 2.33)
**Many**	871	20	5,698	35	1.96 (1.21, 2.99)	1.97 (1.21, 3.04)
**Sensitivity analysis 3 [b]**						
**Few**	14,697	1,579	136,539	116	1 (reference)	1 (reference)
**Moderate**	972	99	7,624	130	1.08 (0.88, 1.32)	1.17 (0.95, 1.42)
**Many**	871	81	5,290	153	1.30 (1.04, 1.60)	1.41 (1.13, 1.75)
**Sensitivity analysis 4 [b]**						
**Few**	13,450	1,906	123,480	154	1 (reference)	1 (reference)
**Moderate**	884	117	6,904	169	1.09 (0.91, 1.31)	1.13 (0.94, 1.35)
**Many**	795	110	4,590	240	1.56 (1.29, 1.86)	1.55 (1.28, 1.87)
**Sensitivity analysis 5: Number of all perceived annoyances [b]**						
**0**	7,185	1,047	70,584	148	1 (reference)	1 (reference)
**1-2**	6,919	944	58,194	162	1.10 (1.01, 1.20)	1.08 (0.99, 1.19)
**≥3**	952	132	5,720	231	1.58 (1.32, 1.88)	1.70 (1.42, 2.03)
**Sensitivity analysis 6: Number of all perceived annoyances [b]**						

**0**	7,185	1,047	70,584	148	1 (reference)	1 (reference)
**1**	5,334	754	46,680	162	1.11 (1.01, 1.22)	1.09 (0.99, 1.20)
**2**	1,585	190	11,514	165	1.08 (0.92, 1.25)	1.07 (0.91, 1.25)
**3**	599	90	3,770	239	1.68 (1.36, 2.05)	1.81 (1.46, 2.21)
**≥4**	353	42	1,950	215	1.40 (1.02, 1.87)	1.50 (1.09, 2.01)
**Sensitivity analysis 7: Number of perceived annoyances due to noise** [c]						
**0**	12,770	1,837	118,432	155	1 (reference)	1 (reference)
**1**	1,862	229	13,074	175	1.14 (1.00, 1.30)	1.16 (1.01, 1.32)
**2**	421	54	3,020	179	1.14 (0.87, 1.47)	1.23 (0.94, 1.58)
**≥3**	97	18	617	292	1.93 (1.18, 2.94)	2.02 (1.23, 3.08)
**Sensitivity analysis 8: Number of perceived annoyances due to thermal discomfort** [d]						
**0**	13,220	1,910	121,980	157	1 (reference)	1 (reference)
**1**	1,429	160	10,239	156	0.99 (0.84, 1.15)	1.03 (0.88, 1.21)
**2**	364	53	2,129	249	1.62 (1.23, 2.09)	1.75 (1.33, 2.26)
**3**	137	15	795	189	1.17 (0.68, 1.86)	1.40 (0.81, 2.22)
**Sensitivity analysis 9: Number of perceived annoyances due to low levels of light** [e]						
**0**	14,922	2,103	133,821	157	1 (reference)	1 (reference)
**1**	228	35	1,322	265	1.75 (1.26, 2.35)	1.73 (1.24, 2.32)
**Sensitivity analysis 10: Number of perceived annoyances due to odour or stuffy air** [f]						
**0**	14,560	2,051	131,153	156	1 (reference)	1 (reference)
**≥1**	590	87	3,990	218	1.44 (1.16, 1.76)	1.47 (1.18, 1.81)
**Sensitivity analysis 11: Individuals with very good, good and fair self-rated health** [g]						
**Few**	12,871	1,734	119,872	145	1 (reference)	1 (reference)
**Moderate**	848	112	6,620	169	1.17 (0.96, 1.40)	1.22 (1.00, 1.46)
**Many**	747	96	4,266	225	1.54 (1.26, 1.87)	1.57 (1.28, 1.91)

N, number of individuals; PY, person-years; IR, incidence rate; IRR, incidence rate ratio; CI, confidence interval; [a] Weighted for non-response. [b] Adjusted for age, sex, educational level, cohabitation status, smoking status, years lived in residence at baseline, season of enrolment, and calendar

year. [c] Adjusted for age, sex, educational level, years lived in residence at baseline, season of enrolment, and calendar year. [d] Adjusted for age, sex, educational level, years lived in residence at baseline, season of enrolment, and calendar year. [e] Adjusted for age, sex, educational level, house type, year of house construction, years lived in residence at baseline, season of enrolment, and calendar year. [f] Adjusted for age, sex, educational level, smoking status, house type, year of house construction, years lived in residence at baseline, season of enrolment, and calendar year. [g] Adjusted for age, sex, educational level, cohabitation status, smoking status, years lived in residence at baseline, season of enrolment, and calendar year.

References to the supplementary materials

1. Kjøller M, Nielsen NS, Rasmussen NK. Sundheds- & sygelighedsundersøgelsen 2000: interviewskema med svarfordeling [The Danish Health and Morbidity Survey 2000: Interview questionnaire with distributions of answers]. Published online 2001.

2. Bryant E, Morganstein D. Sample size determination for longitudinal surveys. In: ; 1987.

3. Kloster S, Kirkegaard AM, Davidsen M, et al. Patterns of Perceived Indoor Environment in Danish Homes. *International Journal of Environmental Research and Public Health*. 2022;19(18). doi:10.3390/ijerph191811498

4. Flachs EM, Eriksen L, Koch MB, et al. *Sygdomsbyrden i Danmark: Sygdomme*. Sundhedsstyrelsen; 2015.

5. Zbozinek TD, Rose RD, Wolitzky-Taylor KB, et al. Diagnostic overlap of generalized anxiety disorder and major depressive disorder in a primary care sample. *Depression and Anxiety*. 2012;29(12):1065-1071. doi:10.1002/da.22026

6. Packness A, Halling A, Hastrup LH, Simonsen E, Wehberg S, Waldorff FB. Socioeconomic position, symptoms of depression and subsequent mental healthcare treatment: a Danish register-based 6-month follow-up study on a population survey. *BMJ open*. 2018;8(10):e020945.
